# Role and mechanism of cardiac insulin resistance in occurrence of heart failure caused by myocardial hypertrophy

**DOI:** 10.18632/aging.102212

**Published:** 2019-08-28

**Authors:** Liling Zheng, Bingbing Li, Sihuang Lin, Liangcai Chen, Hongmu Li

**Affiliations:** 1 Department of Cardiovascular Surgery, First Hospital of Quanzhou Affiliated to Fujian Medical University, Quanzhou, Fujian, China; 2 Department of Cosmetic Surgery, First Hospital of Fuzhou Affiliated to Fujian Medical University, Fuzhou, Fujian, China

**Keywords:** insulin resistance, hypertrophy, heart failure, mitochondria, oxidation

## Abstract

Cardiac insulin resistance plays an important role in the development of heart failure, but the underlying mechanisms remain unclear. Here, we found that hypertrophic hearts exhibit normal cardiac glucose oxidation rates, but reduced fatty acid oxidation rates, compared to Sham controls under basal (no insulin) conditions. Furthermore, insulin stimulation attenuated insulin’s effects on cardiac substrate utilization, suggesting the development of cardiac insulin resistance. Consistent with insulin resistance, p38-MAPK protein levels were reduced in hypertrophic hearts. By contrast, systemic hyperinsulin-euglycemic clamp indicated normal insulin sensitivity. Finally, electron microscopy revealed severe mitochondrial damage in the hypertrophic myocardium. Our results indicate that that cardiac insulin resistance caused by cardiac hypertrophy is associated with mitochondrial damage and cardiac dysfunction. Moreover, our findings suggest that cardiac insulin resistance is independent of systemic insulin resistance, which is also a risk factor for heart failure.

## INTRODUCTION

Heart failure is a complex clinical syndrome that results from many cardiovascular diseases and is the most common cause of death worldwide [1]. Insulin resistance is also associated with heart failure; it is currently thought that diabetes and insulin resistance cause myocardial ischemia, which leads to ischemic cardiomyopathy and heart failure [2]. Many clinical trials have confirmed that systemic insulin resistance is an independent risk factor for heart failure and cardiovascular death [3]. Recently, Witteles suggested that insulin resistance is associated with heart failure independent of coronary artery disease [4]. Further studies in animal models found that myocardial insulin resistance, which is characterized by a significant decline in myocardial glucose uptake capacity and reductions of both eNOS (endothelial nitric oxide synthase) activity and mitochondrial function, co-occurs with systemic insulin resistance. However, the separate effects of systemic insulin resistance and local myocardial insulin resistance on the development of heart failure, as well as the underlying mechanisms involved, remain unclear [5]. Some researchers have suggested not only that the heart is a target organ of systemic insulin resistance, but also that local myocardial insulin resistance is a distinct risk factor for heart failure. In this study, we investigated the effects of myocardial insulin resistance on the development of heart failure.

## RESULTS

### High insulin-normal glucose clamp test

One group of rats was subjected to abdominal aortic constriction (AAC) surgery, while a second group underwent sham surgery. Twenty weeks later, significant cardiac hypertrophy was observed in the AAC group compared to the Sham group. Both raw heart weight and heart weight normalized to body weight differed between the two groups (P <0.001, Table 1). These data demonstrated that cardiac hypertrophy was successfully induced in the 20-week AAC model. All subsequent experiments were performed 20 weeks post-operation. Fasting blood glucose levels also differed between the AAC and Sham groups (P <0.05), but levels for both groups were within the normal range (Table 1). During the insulin clamp experiment, GIR did not significantly differ between the AAC and Sham groups (P > 0.05), suggesting that 20 weeks of abdominal aortic coarctation did not induce systemic insulin resistance in the AAC group (Table 1). These data demonstrated that AAC-induced cardiac hypertrophy is not associated with systemic insulin resistance.

**Table 1:** High insulin-normal glucose clamp, body weight, fasting glucose and glucose infusion rate.

	**Sham group**	**AAC group**	**P value**
Weight (g)	440.8±21.3	440.4±20.7	0.049
Heart dry weight (mg)	289.7±25.4	356.2±25.6	0.001*
Heart weight / body weight	0.66±0.05	0.81±0.07	0.001*
Fasting blood glucose mmol / L	5.27±0.46	5.73±0.41	0.015
GIR (mg/kg·min^−1^)	20.25±1.01	19.92±1.61	0.549

* depicts a statistically significant difference between the groups.

### Cardiac function

To evaluate cardiac function, we subjected AAC and Sham group rats to echocardiography 20 weeks post-operation. Heart rate was lower in the AAC group (P < 0.05). Consistent with the heart weight data, left ventricular posterior wall thickness was significantly higher in the AAC group than in the Sham group (P < 0.001). LVIDd, LVIDs, EF, and FS also differed between the groups (P < 0.001), suggesting that AAC impaired left ventricular (LV) systolic and diastolic dysfunction (P < 0.001, Table 2). Isolated perfused heart experiments further confirmed that AAC increased aortic mean pressure, LV systolic pressure, and LV diastolic pressure. Maximal LV systolic pressure was similar in the two groups, but maximal diastolic pressure was lower in the AAC group compared to the Sham group (P < 0.001), suggesting that AAC surgery impaired LV diastolic function (Table 2).

**Table 2:** Abdominal aortic coarctation after 20 weeks of cardiac size and function.

	**Sham group**	**AAC group**	**P value**
Heart rate	412±35	376±33	0.018
LVPW (mm)	0.193±0.012	0.264±0.038	0.001*
LVIDd (cm)	0.63±0.05	0.77±0.05	0.001*
LVIDs (cm)	0.39±0.03	0.50±0.03	0.001*
EF (%)	78.75±4.77	64.00±10.68	0.001*
FS (%)	50.25±6.99	40.50±5.52	0.001*
MAP (mmHg)	122.75±15.17	186.17±12.97	0.001*
LVSP (mmHg)	94.50±12.27	131.5±16.38	0.001*
LVEDP (mmHg)	11.45±0.80	15.23±1.27	0.001*
+dp/dt max	467.67±31.53	471.25±34.99	0.79
-dp/dt max	262.75±26.40	215.08±24.75	0.001*

Data in the table is represented by mean± sd. LV: left ventricle; LVPWS: systolic left ventricular posterior wall thickness; LVIDd: left ventricular end diastolic diameter; LVIDs: left ventricular systolic diameter; EF: cardiac ejection fraction; FS: left ventricular short axis shortening index; MAP: primary mean arterial pressure; LVSP: left ventricular systolic pressure; LVEDP: left ventricular end-diastolic pressure; +dp / dt max: maximal change rate of systolic left ventricular pressure; - dp / dt max: maximal change rate of diastolic left ventricular pressure.

### Glucose uptake and fatty acid oxidation in isolated perfused heart

Myocardial glucose uptake was similar in the AAC and Sham groups (P > 0.05). Although insulin stimulation increased myocardial glucose uptake in both groups (P < 0.001), this increase was larger in the Sham group than in the AAC group (P < 0.001). Collectively, these data indicated the presence of myocardial insulin resistance in the AAC group (Table 3). Additionally, myocardial fatty acid oxidation was lower in the AAC group than in the Sham group (P < 0.001). While insulin stimulation decreased the fatty acid oxidation rate in both groups, it was reduced to a greater extent in the AAC group than in the control group (P < 0.001, Table 4).

**Table 3:** Myocardial glucose uptake.

	**Sham group**	**AAC group**	**P value**
Basal glucose uptake (μmol/min·g^−1^)	0.74±0.43	0.59±0.19	0.269
Insulin uptake after insulin stimulation (μmol/min·g^−1^)	1.35±0.24	0.93±0.17	0.001*
The increase in glucose uptake after insulin stimulation (μmol/min·g^−1^)	0.61±0.26	0.35±0.22	0.014
Increase in glucose uptake after insulin stimulation (%)	109.33±50.20	55.8±44.02	0.011

**Table 4:** Myocardial fatty acid oxidation.

	**Sham group**	**AAC group**	**P value**
The amount of fatty acid oxidation (μmol/min·g^−1^)	1.00±0.14	0.50±0.08	0.001*
The amount of fatty acid oxidized after insulin stimulation (μmol/min·g^−1^)	0.36±0.07	0.26±0.03	0.001*
The decrease in fatty acid oxidation after insulin stimulation (μmol/min·g^−1^)	0.64±0.14	0.25±0.08	0.001*
Changes in fatty acid oxidation after insulin stimulation (%)	63.13±8.85	42.72±10.90	0.001*

### Expression of p38 MAPK protein in rat myocardium

**Figure 1:**
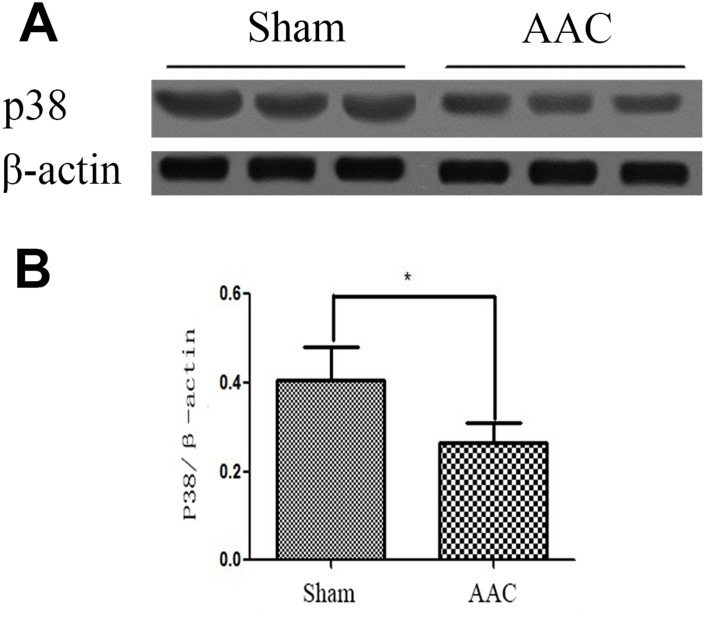
**Expression of p38 MAPK protein in rat myocardium.** Ratios of target p38MAPK protein to internal reference protein β-actin optical density as detected by Western blot are shown.

Western blot indicated that p38 MAPK protein expression was lower in the AAC group than in the Sham group (P < 0.05, Figure 1).

### Myocardial cell ultrastructure

**Figure 2:**
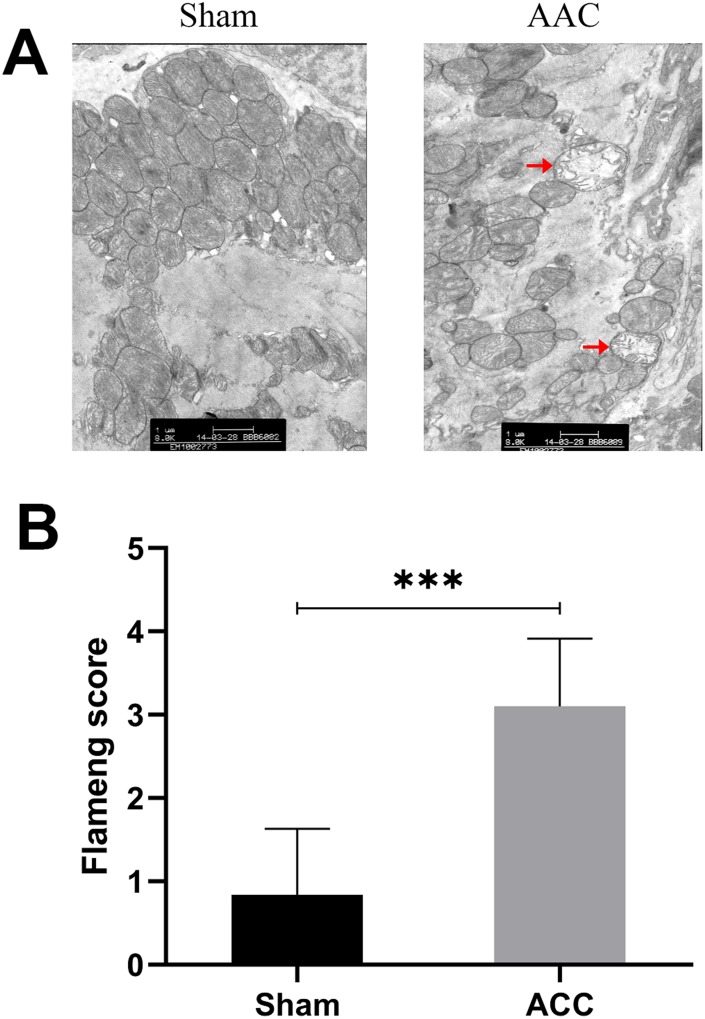
**Myocardial cell ultrastructure.** In sham group myocardia, mitochondrial membranes and matrix particles were typically intact, and the mitochondrial ridge was usually continuous. In AAC group myocardia, mitochondrial swelling, matrix loss, and even vacuolation were observed. The inner and outer membranes were also damaged and incomplete.

While cardiomyocyte ultrastructure was normal in the Sham group, there was obvious mitochondrial damage in AAC hearts. The Flameng score for the Sham group (0.84±0.79) was lower than that in the AAC group (3.10±0.81), indicating that there was more mitochondrial damage in the AAC group than in the Sham group (Figure 2).

## DISCUSSION

Insulin resistance (IR) is characterized by a series of pathophysiological changes that lead to decreased sensitivity to insulin. It affects substrate oxidation and a variety of cell functions, and it can contribute to a variety of diseases. Because insulin resistance is observed not only in type 2 diabetes, but also in a wide variety of diseases and pathological conditions, Reaven developed the concept of “insulin resistance syndrome (IRS) “[6]. In 1995, Stem proposed the “common soil theory,” which posits that insulin resistance is a common risk factor for hypertension, diabetes, heart disease, and stroke [7]. Previous studies of IR have focused on traditional target organs, such as the liver, skeletal muscle, and adipose tissue, but not on the myocardium, which is typically examined only in the context of chronic disease development. However, other studies have shown that myocardium is also an important target tissue for insulin resistance [8].

The following have been proposed as the main mechanisms underlying insulin resistance: (1) genetic variation leading to abnormal insulin structure and biological activity; (2) insulin and post-insulin receptor defects in target tissue; and (3) increases in substances that antagonize the physiological role of insulin, such as catecholamines, growth hormone, glucagon, insulin antibodies, glucocorticoids, and insulin receptor antibodies.

Recent clinical studies have found that IR is an important risk factor and pathologic basis for heart failure, and that it is independent of coronary artery disease. In a previous study on insulin resistance in patients with heart failure, fasting blood glucose levels were similar in controls and chronic heart failure patients, but heart failure patients had higher plasma insulin levels [9]. Animal experiments suggest that myocardial insulin resistance, which involves reductions in myocardial metabolism, insulin-stimulated eNOS activity, and insulin protection effects, co-occurs with systemic IR. However, the mechanisms associated with myocardial insulin resistance and its role in the development of heart failure remain unclear. In this study, we examined insulin sensitivity in the hypertrophic heart to determine whether cardiac insulin resistance is an independent risk factor for heart failure. We found that pressure overload–induced cardiac hypertrophy is associated with myocardial insulin resistance, reduced fatty acid oxidation, and mitochondrial damage. Mechanistically, we found that the reduction of p38 MAPK expression in hypertrophic hearts might impair insulin signaling.

Here, glucose uptake rates (GIR) as measured by high insulin-normal blood glucose clamp did not differ between hypertrophic and normal groups. This indicates that systemic insulin resistance was not induced by cardiac hypertrophy in these rats. Our use of the Langendorff perfused heart model allowed us to examine myocardial insulin resistance *in* *vitro* while excluding the effects of nerves, body fluids, and other systemic factors. The results indicated that insulin-induced glucose uptake was decreased after myocardial hypertrophy; hypertrophy is therefore associated with myocardial insulin resistance.

Insulin-stimulated glucose uptake is widely recognized as a sign of insulin sensitivity, but insulin also plays an important biological role in substrate oxidation processes. For example, insulin promotes glycolysis and glucose oxidation independently from its effects on glucose uptake [10]. Insulin can also activate glycogen synthesis; notably, insulin resistance in patients with type 2 diabetes is characterized by impaired glucose oxidation and glycogen synthesis [11]. Insulin is also involved in the regulation of cell growth and mitochondrial biotransformation-related gene expression, in addition to the acute effects of blood glucose metabolism [12]. We found that insulin-induced decreases in fatty acid oxidation were greater in the AAC group, further linking cardiac hypertrophy to myocardial insulin resistance.

The heart requires a large amount of energy, and glucose and fatty acids provide more than 90% of the energy for ATP synthesis in the heart. When insulin resistance occurs, glucose uptake and utilization is reduced, leading to energy deficiency and cardiac dysfunction. In the AAC group, echocardiography revealed that left ventricular posterior wall thickness increased 20 weeks after abdominal aortic coarctation; together with isolated heart perfusion test results and increased heart weights, these results suggest that the AAC group showed significant cardiac hypertrophy. Furthermore, left ventricular dilatation, ejection fraction, and short axis-shortening index decreased in the AAC group. The maximum left ventricular diastolic pressure change rate was also decreased in the AAC group. In combination with the isolated heart perfusion test, this indicated that left ventricular systolic and diastolic function was impaired in the AAC group (P < 0.001). In addition, mitochondrial granules, rupture, and inner and outer membrane integrity were lost, and other pathological changes were observed in the mitochondria of the myocardium.

The biological effects of insulin are carried out through the signal transduction process and include the following steps: (1) insulin receptor activation; (2) insulin receptor substrate (IRS) phosphorylation; (3) src homology protein binding; and (4) protein kinase and phosphatase signaling cascades [13]. Impairments of any part of the insulin signaling pathway can lead to insulin resistance [14]. We measured p38 protein kinase level and found that p38 MAPK protein expression was significantly lower in hypertrophic myocardium than in the Sham group. Protein kinase p38 is an important member of the MAPK family and plays a key role in mitochondrial biosynthesis. P38 MAPK activates PGC-1 via post-transcriptional regulation and can directly phosphorylate PGC-1 protein, making it active and stable [15]. The main biological function of PGC-1 is to upregulate oxidative metabolism by stimulating myocardial mitochondrial biosynthesis and metabolic gene expression [16]. Reduced p38 protein kinase signal transduction can therefore cause both reduced mitochondrial biosynthesis and oxidative dysfunction.

In summary, we demonstrated in this study that myocardial hypertrophy is associated with myocardial insulin resistance accompanied by disruptions in myocardial mitochondrial function and decreased cardiac systolic function. This cardiac mitochondrial dysfunction, together with decreased fatty acid oxidative capacity, might be caused by decreased p38 MAPK expression. This suggests that myocardial insulin resistance and mitochondrial dysfunction play important roles in the ventricular remodeling and systolic dysfunction that occur in cardiac hypertrophy.

## MATERIALS AND METHODS

### Animals

Sixty male SD rats (body weight 190 ± 10 g) were randomly divided between the AAC (abdominal aortic constriction) group and the Sham group. For the AAC group (heart failure with cardiac hypertrophy, n = 30), heart weight/body weight, left ventricular posterior wall thickness, left ventricular dimension, ejection fraction, and shortening index were measured 20 weeks after surgery; the emergence of dyspnea and pleural effusion performance were also recorded. The same measurements and observations were recorded for Sham group (n = 30) animals 20 weeks after the sham surgery procedure.

### Abdominal aortic constriction

Abdominal aortic constriction was carried out as described by Phrommintikul *et al*. with modifications [17]. The rats were weighed and anesthetized with 10% chloral hydrate (0.3ml/100g). Under sterile conditions, an incision was made along the abdominal midline, 4 cm away from the xiphoid process. The incision was sutured in layers and the animals were injected with penicillin prophylaxis. Sham-operated animals serving as controls were subjected to the same surgical procedure except that the aorta was not constricted. After surgery in a standard animal laboratory laminar flow cabinet, the animals were given fresh drinking water and full price nutritional feed from Shanghai Shi Lake Company for 20 weeks.

### Measurement of cardiac function

Cardiac functions were examined using the Visual Sonics’ Vevo 770TM high-resolution small animal ultrasound system for 20 weeks after operation. Cardiac function was evaluated in EKV mode and M-mode, and the diastolic function of the heart was measured by Doppler’s mitral flow measurement.

### Evaluation of insulin resistance: hyper insulinemic-normal glucose clamp test

All rats were fasted for 12 hours before the hyperinsulinemia-normal blood glucose clamp test was performed. 10% chloral hydrate (0.3ml/100g) was then used for intraperitoneal anesthesia and the bilateral artery and femoral vein were exposed and intubated with silicone rubber catheters (diameter 0.6mm, diameter 1mm); heparin was administered (75 U/kg for initially followed by 25 U/kg per hour) as an anticoagulant. The right femoral artery channel was used for blood glucose measurement, while an infusion pump was used to deliver a constant dose of fast-acting insulin (6.0 mU/kg/min) into the right femoral vein. Basal blood glucose (BBG) levels were measured in a 10 μL sample before infusion. The infusion rate of a 10% glucose solution was adjusted to maintain blood sugar levels close to the initial fasting blood glucose level (BBG ± 0.5 mmol/L), and depth of anesthesia was monitored. The experiment lasted for 120 minutes in total; blood glucose levels and glucose infusion rate (GIR) after 60 minutes were used to establish the insulin sensitivity index for all rats.

### Insulin-stimulated perfusion in isolated hearts

Rats’ hearts were removed 20 weeks after surgery, and the heart perfusion test was performed. Heart rate (HR), left ventricular end diastolic pressure (LVEDP), left ventricular pressure (LVDP), and maximum rate of change (± dp/dt max) of left ventricle systolic and diastolic pressure were measured continuously using the MPA-heart function test system (Shanghai Orcott).

### Rat glucose oxidative and fatty acid oxidative assay

Hearts were isolated and suspended on the Langendorff perfusion system. After 5-10 minutes of regular perfusion, hearts were perfused with KH solution containing glucose and palmitic acid and primed in KH solution with insulin (1 mU/mL) for 30 minutes; the liquid was collected every 5 minutes. 2 mL samples were taken from the effluent of the coronary arteries and centrifuged at 1000 rpm for 5 min. The supernatant was extracted and placed in an EP tube. The cells were labeled with an automatic biochemical analyzer. Free fatty acid and glucose concentration were measured in the perfusion fluid (with or without insulin) before and after cardiac perfusion. Basal glucose and fatty acid oxidation rates and insulin-stimulated changes in those rates and in absolute glucose and fatty acid oxidation levels were calculated. Perfusion liquid volumes were also measured to determine coronary flow rates. After the experiment, hearts were cut and placed in an oven set at 90 ° C for 12 hours to dehydrate completely; the dry weight of the hearts was then measured.

### Measurement of protein kinase p38 protein expression after *in* *vitro* insulin test

Twelve rats were divided into two groups and sacrificed as described in the *in* *vitro* perfusion procedure. The aorta was then intubated and retrograde perfusion of the myocardium was performed using Krebs-Henseleit buffer with glucose and palmitic acid with or without insulin (1mU / mL) to complete *in* *vitro* insulin stimulation and control experiments. The left ventricular myocardium was then placed in liquid nitrogen. p38 MAPK expression was measured in cardiomyocytes of the hypertrophic myocardium by Western blotting.

### Electron microscope analysis of myocardial cell mitochondrial damage

Electron microscopy was performed on the apical myocardium of the Sham and AAC group animals using a PHILIPS EM208 transmission electron microscope. Five fields containing 20 mitochondria each were randomly selected for each group and average mitochondrial Flameng scores were calculated for each set of five fields. Flameng scoring criteria were as follows: 0: normal mitochondria; 1: mild swelling, reduced matrix density, and sputum separation; 2: moderate swelling, severe reduction in matrix density, and separation of sputum; 3: severe swelling with sputum interruption; 4: severe swelling with sputum interruption, internal and external mitochondrial membrane ruptures.

### Statistical analysis

Data are expressed as mean ± standard deviation. Student’s t-test or one-way ANOVAs were performed as appropriate with SPSS 13.0 software. Analysis was performed using GraphPad Prism 5.0 (GraphPad Inc, La Jolla, CA, USA). P < 0.05 was considered statistically significant. P < 0.001 indicated a highly significant difference.
